# Mohs Micrographic Surgery for Dermatofibrosarcoma Protuberans of the Vulva

**DOI:** 10.1155/2009/547672

**Published:** 2009-11-18

**Authors:** K. Doufekas, T. J. Duncan, K. M. Williamson, S. Varma, D. Nunns

**Affiliations:** ^1^Department of Gynaecological Oncology, Nottingham City Hospital, Nottingham NG5 1PB, UK; ^2^Department of Dermatology, Queens Medical Centre, Nottingham NG7 2UH, UK

## Abstract

*Introduction*. Dermatofibrosarcoma Protuberans (DFSP) is a rare cutaneous tumour of low/intermediate malignant potential, which occasionally arises on the vulva. Historically, the treatment has been wide local excision (WLE). Mohs micrographic surgery (MMS) is now recommended to ensure precise margin control. MMS to treat DFSP of the trunk and extremities is well documented. However, no report to date has described its use in vulval DFSP. *Case History*. A 39 year old woman presented with a longstanding nodule in the left labium majus. Histology after surgical removal showed an incompletely excised DFSP. MMS was undertaken with primary closure of the defect. Three years following treatment there is no evidence of recurrence. *Discussion*. The local recurrence rate of DFSP after WLE ranges from 0–75%. Finger-like projections from DFSP into surrounding tissue often results in incomplete excision. Representative vertical sections used in WLE assess less than 1% of the total tumour margin. MMS uses systematic horizontal sectioning. 100% of the tumour margin is microscopically examined. MMS is now advocated to ensure precise margin control.

## 1. Introduction

Dermatofibrosarcoma protuberans (DFSP) is a rare cutaneous tumour of low/intermediate malignant potential, which occasionally arises in the vulva. Historically, the treatment has been wide local excision (WLE). However, local recurrence rates after WLE are high [1]. Microscopic projections of the tumour into surrounding tissue often extend beyond the central nodule, resulting in incomplete excision [2, 3].

Mohs Micrographic Surgery (MMS) offers an alternative therapeutic approach. MMS allows mapping of the extend of DFSP with microscopic examination of its deep and lateral margins. 100% of the tumour margin is examined. The microscopic tumour projections into surrounding tissue are reliably identified and excised. Favourable outcomes are reported with preservation of tissue and a low recurrence rate [1].

MMS to treat DFSP of the trunk and extremities is well documented [1]. However, no report to date has described its use in DFSP of the vulva.

## 2. Case History

A-39-year old woman presented with a longstanding painless nodule in the left labium majus. Histology after surgical removal showed an incompletely excised DFSP. Chest X-Ray showed no evidence of metastasis. 

Further management involved a multidisciplinary team, consisting of a dermatologist, gynaecological oncologist, and histopathologist. 

A residual 10–15 mm nodule was first debulked under local anaesthetic. MMS was then undertaken. A highly specialized and accurate protocol of surgical technique were followed. 

Anatomic orientation of the specimen was defined and drawn onto a map. The specimen was then sliced so that deep and lateral margins lay in the same plane. Horizontal frozen sections were stained with haematoxylin and eosin and examined microscopically to provide a complete 360 degree view of the tumour margins. With MMS the microscopic projections into surrounding tissue can be identified. Residual tumour is then depicted onto the previously drawn anatomic map to guide excision. A further tissue layer is removed and the same meticulous procedure is followed until microscopic examination shows no residual tumour. 

In our patient the tumour was completely removed after one stage of MMS. The defect was repaired with primary closure. Three years following treatment, there is no evidence of recurrence.

## 3. Discussion

### 3.1. Epidemiology

DFSP is a rare neoplasm accounting for 0.1% of all malignancies [4]. It typically occurs between the ages of 20 and 40 years, frequently arising on the trunk. The head and extremities are less commonly involved. 

DFSP of the clitoris and vulva are rare. DFSP of the vulva typically arises in the left labium majus [5]. The reason for this predilection is unclear.

### 3.2. Clinical Features

The tumour often presents as a solid, protuberant nodule. Due to its indolent nature, it often escapes detection in the early stages. Pain and ulceration may occur with tumours in an accelerated growth phase [3]. 

Aggressive local growth is characteristic. MRI can identify the extent of the tumour and its relation to adjacent structures [4]. 

Lymphatic spread of DFSP is rare [2]. Metastatic DFSP is uncommon, occurring in less than 6% of cases. Metastases typically involve the lungs and bones [3].

### 3.3. Pathology

The tumour most probably arises from a stem cell within the dermis. Local growth exhibits fingerlike projections into surrounding tissue. Subsequently, it can invade the subcutaneous tissue, underlying fasciae, muscle and bone [3]. 

Correct histological diagnosis can be problematic as it is often difficult to distinguish DFSP from other fibrohistiocytic neoplasms [3]. Comprehensive immunohistochemical studies aid the diagnosis. 

Histology typically shows elongated fibroblasts with small nuclei arranged in a “storiform” pattern. Compared to fibrous histiocytomas DFSP displays a more uniform appearance with little nuclear pleomorphism and low to moderate mitotic activity. Absence of necrosis and immunohistochemical staining for CD34 differentiate DFSP from fibrous histiocytomas [3].

### 3.4. Treatment

Historically, the treatment for DFSP has been wide local excision (WLE) of both primary and recurrent lesions.

The local recurrence rate of DFSP after WLE ranges from 0%–21% for trunk sites and 50%–75% for head and neck sites [1]. In the vulva the local recurrence rate after WLE ranges from 20%–49% [2]. Microscopic projections of the tumour beyond the central nodule often result in incomplete excision. Representative vertical sections used in WLE assess less than 1% of the total tumour margin. There is hence an increased risk of potentially missing these narrow projections, which may lead to tumour recurrence. 

MMS uses systematic horizontal sectioning with 100% of the tumour margin being microscopically examined. This increases the likelihood of any tumour projections being detected.

Since MMS allows for accurate margin control, maximum preservation of healthy tissue with complete excision can be achieved. It, therefore, leaves the smallest post surgical defect and minimises recurrence. Nearly 75% of such wounds can be managed with primary closure. 

Tissue conservation was important in our case to preserve function, with the DFSP being close to the clitoris. Wide local excision with 3 cm margins as previously recommended [2] may have been unduly mutilating.

MMS is now advocated to ensure precise margin control. The favorable success rate of MMS compared to WLE in patients with DFSP is well documented [1]. Site distribution for this technique has included head and neck, extremities, and the clitoris but not the vulva.

The local recurrence rate after the first MMS treatment for all patients with DFSP, has been reported as 7% over a 5-year period. 5 year cure rates have been reported as 92% for head and neck sites, 94% for trunk sites and 100% for DFSP in extremity sites. The 5-year cure rate for all sites was 93% after the first MMS treatment and 98.5% after the second [1].

Local recurrence after MMS may be related to “skip” areas of tumour that become separated from the main tumour mass. However 25% of local-recurrences develop after 5 years [1]. Long term follow up is recommended.

## 4. Conclusion

WLE for DFSP of the vulva has previously been described. MMS for DFSP of the vulva represents, however, a novel treatment. Treatment outcomes for DFSP at other sites are uniformly more favorable with MMS when compared with standard WLE [1]. This case also highlights the utility of Mohs Surgery in conserving normal tissue.

We, therefore, propose that MMS is considered in all cases of primary or recurrent DFSP of the vulva.
